# Determinants of unsuccessful treatment outcomes and mortality among tuberculosis patients in Malaysia: A registry-based cohort study

**DOI:** 10.1371/journal.pone.0231986

**Published:** 2020-04-22

**Authors:** Peter Seah Keng Tok, Su May Liew, Li Ping Wong, Asmah Razali, Tharani Loganathan, Karuthan Chinna, Nurhuda Ismail, Naim Abdul Kadir

**Affiliations:** 1 Department of Social and Preventive Medicine, Centre for Epidemiology and Evidence-Based Practice, Faculty of Medicine, University of Malaya, Kuala Lumpur, Malaysia; 2 Institute for Clinical Research, National Institutes of Health (NIH), Ministry of Health Malaysia, Bandar Setia Alam, Shah Alam, Selangor, Malaysia; 3 Department of Primary Care Medicine, Faculty of Medicine, University of Malaya, Kuala Lumpur, Malaysia; 4 Sector of TB/Leprosy, Disease Control Division, Ministry of Health Malaysia, Federal Government Administration Centre, Putrajaya, Malaysia; 5 School of Medicine, Faculty of Health & Medical Sciences, Taylor’s University, Subang Jaya, Selangor, Malaysia; 6 Department of Public Health Medicine, Faculty of Medicine, Universiti Teknologi MARA, Sungai Buloh, Selangor, Malaysia; Jamia Hamdard, INDIA

## Abstract

**Introduction:**

The monitoring of tuberculosis (TB) treatment outcomes and examination of the factors affecting these outcomes are important for evaluation and feedback of the national TB control program. This study aims to assess the TB treatment outcomes among patients registered in the national TB surveillance database in Malaysia from 2014 until 2017 and identify factors associated with unsuccessful treatment outcomes and all-cause mortality.

**Materials and methods:**

Using registry-based secondary data, a retrospective cohort study was conducted. TB patients’ sociodemographic characteristics, clinical disease data and treatment outcomes at one-year surveillance were extracted from the database and analyzed. Logistic regression analysis was used to determine factors associated with unsuccessful treatment outcomes and all-cause mortality.

**Results:**

A total of 97,505 TB cases (64.3% males) were included in this study. TB treatment success (cases categorized as cured and completed treatment) was observed in 80.7% of the patients. Among the 19.3% patients with unsuccessful treatment outcomes, 10.2% died, 5.3% were lost to follow-up, 3.6% had outcomes not evaluated while the remaining failed treatment. Unsuccessful TB treatment outcomes were found to be associated with older age, males, foreign nationality, urban dwellers, lower education levels, passive detection of TB cases, absence of bacille Calmette-Guerin (BCG) scar, underlying diabetes mellitus, smoking, extrapulmonary TB, history of previous TB treatment, advanced chest radiography findings and human immunodeficiency virus (HIV) infection. Factors found associated with all-cause mortality were similar except for nationality (higher among Malaysians) and place of residence (higher among rural dwellers), while smoking and history of previous TB treatment were not found to be associated with all-cause mortality.

**Conclusions:**

This study identified various sociodemographic characteristics and TB disease-related variables which were associated with unsuccessful TB treatment outcomes and mortality; these can be used to guide measures for risk assessment and stratification of TB patients in future.

## Introduction

An estimated 10 million new tuberculosis (TB) cases were diagnosed worldwide in 2018. Within the same year, 1.5 million people died from TB. Despite high cure rates that are achievable with timely diagnosis and appropriate antibiotics treatment, TB remains as one of the top ten causes of death globally and is the leading cause of death from a single infectious agent [1].

Strategies for TB control needs to be tailored to diverse country settings. A thorough knowledge of a specific country’s TB disease epidemiology is essential to map out a comprehensive national strategic plan for TB [2]. Monitoring of TB treatment outcomes through patients’ cohort analyses is important to evaluate and provide feedback on national TB control programmes [3]. One of the key indicator for monitoring implementation of the End TB Strategy by the World Health Organization (WHO) is the TB treatment success rate, with the recommended target level of ≥90% by 2025 at the latest [2, 4].

Malaysia has an intermediate TB burden, with an estimated total TB incidence of 92 (79–106) per 100,000 population in 2018 [5]. Over the last decade, TB control services in Malaysia had been strengthened; these include efforts in upgrading TB recording and reporting system as well as reinforcing the network of public health laboratories [6]. The TB treatment success rates for incident TB cases in Malaysia had, accordingly, gradually climbed from 76% in the year 2013 to 81% in the year 2017 patients’ cohort [1, 5, 7]. The rates, however, remained below the recommended target of ≥90%.

Worldwide, studies had investigated on TB treatment outcomes by looking into factors that were associated with unsuccessful treatment outcomes as well as mortality among TB patients [8–13]. Important predictive factors reported include patients’ sociodemographic characteristics (age, sex, education levels, employment), underlying comorbidities such as diabetes mellitus (DM) and human immunodeficiency virus (HIV) infection as well as TB disease profile/factors (sputum and radiological findings, site of TB infection, history of previous TB treatment).

Findings from studies investigating on TB treatment outcomes invariably differ according to the local settings of TB patients’ population [8–13]. It is therefore apt to perform cohort analyses in the local context, from time to time, to monitor the TB treatment outcomes and provide feedback on the national TB control program [3, 14]. In Malaysia, previous studies investigating on TB treatment outcomes were often healthcare-center-based and focused on the different subpopulations of TB patients [14–17]. A previous study was performed using the year 2012 national TB patients’ cohort but had its limitation in the amount of missing information [9]. Since then, efforts to improve data recording and documentation were rolled out nationwide to improve the quality of information in the national TB database.

The present study, therefore, aims to provide updated and comprehensive evidence on TB treatment outcomes in Malaysia by looking at the outcomes in the most recent national cohorts of TB patients in Malaysia (from year 2014 until year 2017) and investigate factors associated with unsuccessful TB treatment outcomes as well as all-cause mortality.

## Materials and methods

### Study design and data collection

This was a retrospective cohort study of TB patients in Malaysia from the year 2014 until year 2017, utilizing data from the national TB surveillance database. TB is a notifiable disease by law in Malaysia, under Act 342: Prevention and Control of Infectious Diseases [18]. In Malaysia, TB diagnosis is supported by imaging and laboratory tests, and is confirmed by isolation of *Mycobacterium tuberculosis* from patients’ clinical samples. Acid fast bacilli (AFB) detection on smears and cultures from patients’ clinical specimens are routinely performed to establish TB diagnosis. Molecular methods for TB detection are not part of routine diagnostic investigation due to the high cost and requirement for sophisticated laboratory infrastructure.

Once diagnosed with TB, the recording and reporting of patients’ information, socio-demographic characteristics, medical history/co-morbidities as well as their TB disease condition throughout the treatment period, are performed by healthcare professionals at the respective healthcare facilities. Malaysia uses standardized TB recording and reporting system for every individual TB patient, and this had been incorporated into the national electronic TB surveillance database since the year 2013 [6, 19].

Data in the surveillance database were consolidated at the national level and TB cases from all states in Malaysia were included in the study. In this study, we included all registered TB cases from the year 2014 until year 2017 which had their TB treatment outcomes defined at one-year surveillance. Cases which changed diagnosis (referring to cases which were previously provisionally diagnosed as TB but were later ruled out) as well as cases with unknown treatment outcome at one-year surveillance (this includes cases with missing information on treatment outcome and cases which were still on treatment), were excluded. We also excluded all cases with multidrug-resistant TB (MDR-TB) as the treatment outcomes definition for MDR-TB cases are different and as recommended in the WHO reporting framework, these cases should be excluded from the main TB cohort when calculating treatment outcomes [20].

### Operational definition

TB treatment outcomes were defined and recorded at one-year surveillance. According to the Clinical Practice Guidelines for Management of Tuberculosis by Ministry of Health, Malaysia [21] and the definitions and reporting framework for TB by WHO [20], the following treatment outcome operational terms were used for this study:

1. Cured: Bacteriologically confirmed TB patient who was subsequently smear- or culture- negative during the last month of treatment or on at least one previous occasion.
2. Completed treatment: Patient who completed TB treatment without meeting the criteria for cure or treatment failure.
3. Treatment failed: Tb patient whose sputum smear or culture was positive at five months or later during TB treatment.
4. Died (Mortality): TB patient who dies for any reason before starting or during the course of TB treatment (all-cause mortality).
5. Loss to follow-up: TB patient who did not start treatment or whose treatment was interrupted for two consecutive months or more.
6. Not evaluated: TB patient with no assigned treatment outcome. In our study, this included TB cases who were “transferred out” to another country for whom the treatment outcomes were not known.

Treatment success or favorable treatment outcomes was defined as the sum of cases who were cured and completed treatment whereas all the other outcomes (treatment failed, died, loss to follow-up and not evaluated) were considered unsuccessful or unfavorable treatment outcomes.

Definitions for the categories of TB cases according to their history of previous TB treatment were based on the WHO definitions and reporting framework [20]. Briefly, new patients were patients who had never been treated for TB or had taken anti-TB drugs for less than one month, while patients who had previously been treated were categorized based on the treatment outcome of their most recent course of treatment: those who were cured or completed treatment were defined as relapse patients, those who failed treatment were defined as treatment after failure patients while those who were lost to follow-up were defined as treatment after loss to follow-up patients.

Based on the anatomical site of TB disease, all cases were classified into pulmonary TB (PTB) or extrapulmonary TB (EPTB). Cases with both PTB and EPTB involvement were classified as PTB. Miliary TB cases were also classified as PTB as they had lung lesions. This is in accordance with the classification outlined in the WHO definition and reporting framework [20].

### Statistical analysis

Data were analyzed using IBM SPSS version 20.0 (IBM Corporation, Armonk, NY, USA). Sociodemographic characteristics of TB patients and their disease profile, including treatment outcomes, were summarized in frequencies and percentages for categorical variables and in appropriate measures of central tendency and dispersion for continuous variables. Multiple imputation by fully conditional specification using an iterative Markov chain Monte Carlo (MCMC) method was used to account for missing data in variables of interest [22, 23]. Univariate and multivariate binary logistic regression analyses were used to examine the associations between selected sociodemographic characteristics and TB disease profile variables with outcomes of interest (unsuccessful treatment outcome and all-cause mortality). The crude odds ratios (OR) and adjusted odds ratios (AOR), with their corresponding 95% confidence interval (CI) and p-values, were reported for each independent variable of interest. In all tests, a p-value of <0.05 was considered to be statistically significant.

### Ethical considerations

This study utilized secondary data with personal information and patient identifiers removed. All cases included were anonymized and thus written informed consent from individual patients were not sought. The study was approved by the Medical Research and Ethics Committee (MREC), Ministry of Health, Malaysia (NMRR-18-3399-45073).

## Results

A total of 100,754 cases were registered in the national TB surveillance database from the year 2014 until year 2017. After excluding cases with unknown treatment outcomes (n = 740) and cases which changed diagnosis (n = 2195), there were 97,819 confirmed TB cases with assigned treatment outcomes at one-year surveillance. We then further excluded 314 cases which were found to be MDR-TB, bringing to a final total of 97,505 TB cases to be included for analysis.

### Baseline characteristics and treatment outcomes

Table 1 illustrates the sociodemographic characteristics of all cases included in the study (N = 97,505). The mean age for all cases was 42.7 years (SD 18.14). Only 2.9% of the patients were below 15 years old. Male patients (64.3%) outnumbered female patients, and around one in eight (12.7%) of the patients were non-Malaysians. Most of the non-Malaysian cases (>90%) originated from the Philippines (34.4%), Indonesia (33.8%), Myanmar (14.8%), Nepal (5.3%) and Bangladesh (3.4%).

**Table 1:** Sociodemographic characteristics of the study population (n = 97,505).

Characteristics		*n* (%)
**Patient age groups (*n* = 97,503)**		
	Below 5 years old	1,049 (1.1)
	5 to 14 years old	1,766 (1.8)
	15 to 24 years old	15,210 (15.6)
	25 to 34 years old	18,684 (19.2)
	35 to 44 years old	16,513 (16.9)
	45 to 54 years old	16,502 (16.9)
	55 to 64 years old	14,796 (15.2)
	65 years old and above	12,983 (13.3)
**Gender (*n* = 97,505)**		
	Female	34,845 (35.7)
	Male	62,660 (64.3)
**Nationality (*n* = 97,505)**		
	Malaysian	85,158 (87.3)
	Non-Malaysian	12,347 (12.7)
**Location of residence (*n* = 96,825)**		
	Urban	53,392 (55.1)
	Rural	43,433 (44.9)
**Education level (*n* = 96,581)**		
	Tertiary	12,780 (13.2)
	Secondary	47,851 (49.5)
	Primary	17,766 (18.4)
	No formal education	18,184 (18.8)

Table 2 provides the TB disease profile and treatment outcomes of the cases in the study cohort. The majority of the cases were new TB cases (92.5%), had pulmonary TB involvement (87.0%) and were sputum smear positive (65.9%). Among cases with known HIV infection status, 6.0% of them were HIV positive. Treatment success, or favorable treatment outcomes, were recorded in 80.7% of the patients. Approximately one in ten patients (10.2%) died during the one year surveillance. Cases which were loss to follow-up (5.3%), not evaluated including transferred out cases (3.6%), and failed treatment (0.1%) made up the remaining unsuccessful treatment outcomes. Of the 3,536 cases which were not evaluated including transferred out, 3,313 cases (93.7%) were of foreign nationality (non-Malaysians).

**Table 2:** TB disease profile and outcomes of the study population (n = 97,505).

Disease profile and outcomes		*n* (%)
**TB case detection (*n* = 97,505)**		
	Passive	89,086 (91.4)
	Active or screening	8,419 (8.6)
**BCG scar (*n* = 97,505)**		
	Yes	79,699 (81.7)
	No	17,806 (18.3)
**Diabetes mellitus (*n* = 97,505)**		
	No	79,954 (82.0)
	Yes	17,551 (18.0)
**Smoking (*n* = 97,505)**		
	No	66,222 (67.9)
	Yes	31,283 (32.1)
**TB site (*n* = 97,505)**		
	Pulmonary TB	84,814 (87.0)
	Extrapulmonary TB	12,691 (13.0)
**TB case category (*n* = 97,505)**		
	New case	90,225 (92.5)
	Relapse case	4,838 (5.0)
	Treatment after failure	208 (0.2)
	Treatment after loss to follow-up	2,234 (2.3)
**Sputum smear (*n* = 93,732)**		
	Positive	61,797 (65.9)
	Negative	31,935 (34.1)
**Chest radiography (*n* = 95,617)**		
	No lesion	10,850 (11.3)
	Minimal	48,359 (50.6)
	Moderately advanced	32,593 (34.1)
	Far advanced	3,815 (4.0)
**HIV infection (*n* = 92,764)**		
	Negative	87,161 (94.0)
	Positive	5,603 (6.0)
**TB treatment outcomes (all outcomes) (*n* = 97,505)**		
	Cured	48,682 (49.9)
	Treatment completed	30,018 (30.8)
	Treatment failed	145 (0.1)
	Died	9,963 (10.2)
	Loss to follow-up	5,161 (5.3)
	Not evaluated	3,536 (3.6)
**TB treatment outcomes (grouped) (*n* = 97,505)**		
	Favorable	78,700 (80.7)
	Unfavorable	18,805 (19.3)

TB, tuberculosis; BCG, bacille Calmette-Guerin; HIV, human immunodeficiency virus.

### Factors associated with unsuccessful treatment outcomes

The results from logistic regression analysis on the factors associated with unsuccessful TB treatment outcomes are presented in Table 3. Among the tested variables, older age, males, foreign nationality (non-Malaysians), urban location of residence, lower education levels, passive detection of TB cases absence of bacille Calmette-Guerin (BCG) scar, underlying DM, smoking, extrapulmonary involvement or EPTB, history of previous TB treatment, advanced chest radiography (CXR) findings, and HIV positivity were statistically significant. Sputum smear findings were not found to be associated with unsuccessful TB treatment outcomes.

**Table 3:** Univariable and multivariable analysis for unsuccessful treatment outcomes.

Variables		Unsuccessful outcomes *n* (%)	Univariate OR (95% CI)	*P value*	Multivariate (*n =* 97,505) AOR (95% CI)	*P value*
**Age, in years (*n* = 97,503)**						
	Mean difference ± SE	3.586 ± 0.147	1.011 (1.010, 1.012)	<0.001	1.015 (1.014, 1.016)	<0.001
**Sex (*n* = 97,505)**						
	Female	4,837 (13.9)	1		1	
	Male	13,968 (22.3)	1.78 (1.72, 1.84)	<0.001	1.47 (1.41, 1.53)	<0.001
**Nationality (*n* = 97,505)**						
	Malaysian	14,310 (16.8)	1		1	
	Non-Malaysian	4,495 (36.4)	2.83 (2.72, 2.95)	<0.001	2.94 (2.77, 3.12)	<0.001
**Location of residence (*n* = 96,825)**						
	Urban	10,660 (20.0)	1.11 (1.07, 1.14)	<0.001	1.21 (1.17, 1.26)	<0.001
	Rural	7,992 (18.4)	1		1	
**Education level (*n* = 96,581)**						
	Tertiary	1,217 (9.5)	1		1	
	Secondary	8,350 (17.5)	2.01 (1.89, 2.14)	<0.001	1.67 (1.56, 1.79)	<0.001
	Primary	3,809 (21.4)	2.59 (2.42, 2.78)	<0.001	1.81 (1.67, 1.95)	<0.001
	No formal education	5,078 (27.9)	3.68 (3.44, 3.94)	<0.001	2.04 (1.89, 2.20)	<0.001
**TB case detection (*n* = 97,505)**						
	Passive	17,317 (19.4)	1.12 (1.06, 1.19)	<0.001	1.15 (1.08, 1.22)	<0.001
	Active or screening	1,488 (17.7)	1		1	
**BCG scar (*n* = 97,505)**						
	Yes	13,380 (16.8)	1		1	
	No	5,425 (30.5)	2.17 (2.09, 2.25)	<0.001	1.21 (1.15, 1.27)	<0.001
**Diabetes mellitus (*n* = 97,505)**						
	No	15,383 (19.2)	1		1	
	Yes	3,422 (19.5)	1.02 (0.98, 1.06)	0.433	1.08 (1.03, 1.13)	0.002
**Smoking (*n* = 97,505)**						
	No	11,530 (17.4)	1		1	
	Yes	7,275 (23.3)	1.44 (1.39, 1.49)	<0.001	1.08 (1.04, 1.13)	<0.001
**TB site (*n* = 97,505)**						
	Pulmonary TB	16,467 (19.4)	1		1	
	Extrapulmonary TB	2,338 (18.4)	0.94 (0.89, 0.98)	0.008	1.31 (1.22, 1.41)	<0.001
**TB case category (*n* = 97,505)**						
	New case	16,735 (18.5)	1		1	
	Relapse case	1,107 (22.9)	1.30 (1.22, 1.40)	<0.001	1.17 (1.09, 1.26)	<0.001
	Treatment after failure	69 (33.2)	2.18 (1.63, 2.91)	<0.001	2.17 (1.60, 2.94)	<0.001
	Treatment after loss to follow-up	894 (40.0)	2.93 (2.69, 3.19)	<0.001	2.85 (2.60, 3.12)	<0.001
**Sputum smear (*n* = 93,732)**						
	Positive	12,091 (19.6)	1		1	
	Negative	5,951 (18.6)	0.94 (0.91, 0.98)	0.001	1.00 (0.96, 1.04)	0.959
**Chest radiography (*n* = 95,617)**						
	No lesion	1,765 (16.3)	1		1	
	Minimal	8,061 (16.7)	1.03 (0.97, 1.09)	0.309	1.06 (0.99, 1.15)	0.103
	Moderately advanced	7,286 (22.4)	1.48 (1.40, 1.57)	<0.001	1.45 (1.34, 1.56)	<0.001
	Far advanced	1,340 (35.1)	2.79 (2.56, 3.03)	<0.001	2.68 (2.42, 2.96)	<0.001
**HIV infection (*n* = 92,764)**						
	Negative	14,707 (16.9)	1		1	
	Positive	2,426 (43.3)	3.76 (3.56, 3.98)	<0.001	4.26 (4.01, 4.53)	<0.001

OR, odds ratio; AOR, adjusted odds ratio; SE, standard error (of the mean difference); TB, tuberculosis; BCG, bacille Calmette-Guerin; HIV, human immunodeficiency virus.

### Factors associated with all-cause mortality

The results from logistic regression analysis on the factors associated with all-cause mortality are presented in Table 4. Among the tested variables, older age, males, Malaysians (nationality), rural location of residence, lower education levels, passive detection of TB case, absence of BCG scar, underlying DM, extrapulmonary involvement or EPTB, advanced CXR findings, and HIV positivity were statistically significant. Smoking, history of previous TB treatment and sputum smear findings were not found to be associated with all-cause mortality.

**Table 4:** Univariable and multivariable analysis for all-cause mortality.

Variables		Unsuccessful outcomes *n* (%)	Univariate OR (95% CI)	*P value*	Multivariate (*n =* 97,505) AOR (95% CI)	*P value*
**Age, in years (*n* = 97,503)**						
	Mean difference ± SE	12.190 ± 0.188	1.039 (1.037, 1.040)	<0.001	1.042 (1.040, 1.043)	<0.001
**Sex (*n* = 97,505)**						
	Female	2,730 (7.8)	1		1	
	Male	7,233 (11.5)	1.54 (1.47, 1.61)	<0.001	1.12 (1.06, 1.19)	<0.001
**Nationality (*n* = 97,505)**						
	Malaysian	9,130 (10.7)	1.66 (1.54, 1.79)	<0.001	1.15 (1.04, 1.26)	0.005
	Non-Malaysian	833 (6.7)	1		1	
**Location of residence (*n* = 96,825)**						
	Urban	4,919 (9.2)	1		1	
	Rural	4,976 (11.5)	1.28 (1.22, 1.33)	<0.001	1.13 (1.08, 1.19)	<0.001
**Education level (*n* = 96,581)**						
	Tertiary	656 (5.1)	1		1	
	Secondary	4,537 (9.5)	1.94 (1.78, 2.11)	<0.001	1.39 (1.27, 1.52)	<0.001
	Primary	2,440 (13.7)	2.94 (2.69, 3.22)	<0.001	1.50 (1.36, 1.65)	<0.001
	No formal education	2,254 (12.4)	2.62 (2.39, 2.86)	<0.001	1.73 (1.56, 1.91)	<0.001
**TB case detection (*n* = 97,505)**						
	Passive	9,217 (10.3)	1.19 (1.10, 1.28)	<0.001	1.21 (1.11, 1.32)	<0.001
	Active or screening	746 (8.9)	1		1	
**BCG scar (*n* = 97,505)**						
	Yes	7,793 (9.8)	1		1	
	No	2,170 (12.2)	1.28 (1.22, 1.35)	<0.001	1.07 (1.00, 1.14)	0.037
**Diabetes mellitus (*n* = 97,505)**						
	No	7,499 (9.4)	1		1	
	Yes	2,464 (14.0)	1.58 (1.50, 1.66)	<0.001	1.22 (1.15, 1.28)	<0.001
**Smoking (*n* = 97,505)**						
	No	6,313 (9.5)	1		1	
	Yes	3,650 (11.7)	1.25 (1.20, 1.31)	<0.001	0.97 (0.92, 1.02)	0.190
**TB site (*n* = 97,505)**						
	Pulmonary TB	8,658 (10.2)	1		1	
	Extrapulmonary TB	1,305 (10.3)	1.01 (0.95, 1.07)	0.796	1.47 (1.34, 1.61)	<0.001
**TB case category (*n* = 97,505)**						
	New case	8,997 (10.0)	1		1	
	Relapse case	675 (14.0)	1.46 (1.35, 1.59)	<0.001	1.04 (0.95, 1.14)	0.404
	Treatment after failure	24 (11.5)	1.18 (0.77, 1.80)	0.452	1.10 (0.70, 1.72)	0.684
	Treatment after loss to follow-up	267 (12.0)	1.23 (1.08, 1.40)	0.002	1.08 (0.94, 1.24)	0.271
**Sputum smear (*n* = 93,732)**						
	Positive	6,191 (10.0)	1		1	
	Negative	3,347 (10.5)	1.05 (1.01, 1.10)	0.026	1.04 (0.99, 1.10)	0.133
**Chest radiography (*n* = 95,617)**						
	No lesion	930 (8.6)	1		1	
	Minimal	3,822 (7.9)	0.92 (0.85, 0.99)	0.021	1.05 (0.95, 1.16)	0.332
	Moderately advanced	4,087 (12.5)	1.53 (1.42, 1.65)	<0.001	1.75 (1.58, 1.94)	<0.001
	Far advanced	949 (24.9)	3.53 (3.20, 3.90)	<0.001	4.50 (3.98, 5.10)	<0.001
**HIV infection (*n* = 92,764)**						
	Negative	7,426 (8.5)	1		1	
	Positive	1,748 (31.2)	4.87 (4.58, 5.18)	<0.001	7.47 (6.97, 8.00)	<0.001

OR, odds ratio; AOR, adjusted odds ratio; SE, standard error (of the mean difference); TB, tuberculosis; BCG, bacille Calmette-Guerin; HIV, human immunodeficiency virus.

## Discussion

In this retrospective cohort study, we looked at factors associated with unsuccessful TB treatment outcomes and all-cause mortality among TB patients in Malaysia from 2014 until 2017. During this period, almost one in five TB patients (19.3%) had an unsuccessful TB treatment outcome, with the most common being all-cause mortality (10.2%).

The rate of mortality remained similar to that observed back in 2012 by a previous local study [9]. Following findings from the previous study, efforts such as high-risk group screening had been initiated nationwide with aims to detect TB early and prevent its complications, but the impact of these efforts are not yet seen in the present study. Therefore, it is important to relook into the factors associated with all-cause mortality to optimize patients’ risk stratification and clinical management.

TB cases which were lost to follow-up (5.3% of the total cohort) also warrant significant concern as they are at higher risk for reactivation of active TB, as well as developing MDR-TB [24, 25]. Loss to follow-up, or default, is influenced by a myriad of interrelated factors such as patients’ beliefs and personal factors, health system and service factors, economics including poverty and gender discrimination as well as the social context in terms of support and TB related stigma [26–28]. Many of these factors are not captured quantitatively in routine surveillance data and is therefore beyond the scope of our study. We propose incorporating qualitative input in the future for further exploration of loss to follow-up among TB patients in the local context.

For TB cases which were not evaluated including transferred out to another country (3.6% of the total cohort), the majority of them were of foreign nationality with most cases coming from neighboring countries such as Indonesia and the Philippines [6]. In Malaysia, foreign workers with active TB are usually repatriated within a month [9, 29]. After they were repatriated, their treatment continuation and eventual treatment outcomes were often unable to be evaluated. Efforts to address this should look into establishing a system of cross-border (national border or boundary) referral or collaboration to improve TB prevention and management, including ensuring continuity of care to achieve favorable TB treatment outcomes [30, 31].

TB cases in our study cohort were mostly adult males, a trend similarly observed worldwide [1]. We also found that being males and older age were significantly associated with unsuccessful TB treatment outcomes and all-cause mortality, concurring with findings reported elsewhere [12, 32, 33]. Urban and rural places of residence, as well as varying levels of education, were well represented with fair distribution among patients in our study cohort. Therefore, similar to what is being observed globally [1], TB disease can affect anyone anywhere in Malaysia.

We found that rural TB cases were at higher odds for mortality, while lower levels of education were associated with both unsuccessful treatment outcomes and mortality. A similar association with unsuccessful treatment outcomes had been reported elsewhere [13, 32, 34]. While rural areas of residence were associated with all-cause mortality, TB cases who were urban dwellers had higher odds for unsuccessful treatment in general. This was because there were higher rates of loss to follow-up and outcomes not evaluated (including transferred out) patients among urban dwellers, both of which were added collectively into the unsuccessful treatment outcomes. These observations are important as they provide leverage points and areas for TB control and prevention efforts to be focused on.

The study findings show the odds of unsuccessful treatment outcomes among TB patients who were HIV positive to be at least four times more, and the odds of all-cause mortality to be at least seven times more, when compared to TB patients who were HIV negative. This reinforces findings from previous local and worldwide studies [9, 35, 36]. Recognizing this dual burden of TB and HIV, the WHO had advocated and emphasized the need delivering integrated TB and HIV services, and provided guideline on collaborative TB/HIV activities for national programs and other stakeholders [37]. In Malaysia, TB/HIV collaborative activities had been initiated, and enhancement of these activities is among the strategies outlined in the national strategic plan for TB control [6].

Absence of BCG scar, underlying DM, EPTB and advanced CXR findings had also been found to be associated with both unsuccessful TB treatment outcomes and mortality. These findings were similar to those previously reported in a local study except for co-morbidity with DM, which was found to be not significant back then [9]. Improved surveillance efforts over the recent years to detect and report DM among TB patients (and vice versa) may have contributed towards uncovering this association in the present study. Worldwide, studies had found that DM increases the risk for developing TB, reactivates dormant TB, and worsens the clinical course of TB [38, 39]. A collaborative framework for care and control of TB and DM is therefore crucial given the rising prevalence of diabetes in many countries worldwide, including Malaysia [1, 39, 40].

We found smoking to be associated with unsuccessful TB treatment outcomes, but not mortality. The nature of data collection for smoking in this study, which was self-reporting by patients, and the all-cause mortality used (instead of TB-related death) may have affected these findings. There is consistent evidence worldwide that tobacco smoking is associated with increased risk of active TB, poor TB treatment outcomes as well as TB mortality [41, 42]. Smoking cessation efforts, therefore, should be included in standard practice guidelines for TB case management [43, 44].

Our study draws strength from recruiting all registered TB cases in the national surveillance database in the most recent years where complete treatment outcomes are available, totaling to more than 90,000 patients. Less than 5% missing observations were noted for variables age, location of residence, education level, sputum smear, CXR findings, and HIV infection; these were imputed accordingly.

A limitation of our study is that it was conducted retrospectively using secondary data from surveillance database. As such, certain variables such as socio-economic factors (employment, profession, income), existing co-morbidities other than DM and HIV, malnutrition and risk behavior (alcohol and drug abuse) were not readily available for investigation. We hope that future studies could look into investigating these parameters as well as encourage efforts to start capturing these variables routinely in the surveillance database for all TB cases.

Over the recent years, strategic interventions and key activities had been planned and rolled out under the National Strategic Plan for TB Control in Malaysia (2016–2020) to improve TB treatment outcomes [6]–these include efforts to enhance TB case detection, TB/HIV collaborative activities and management of co-morbidities. At present, efforts are ongoing to monitor the impact of these efforts and subsequently plan for the next NSP from year 2021 onwards.

Our study findings, however, showed that not much has changed since the previous study conducted in 2012 [9]. While this could be because the impact of the strategic interventions carried out so far may require more time to be discernible, findings from our study are important to provide feedback to the existing TB control program and guide future measures to ensure that we remain on the right track.

Findings from this study add on to the existing evidence on factors associated with unsuccessful TB treatment outcomes. Moving forward, we need to engage all relevant stakeholders to identify and subsequently, focus on important factors to be acted upon. Planned implementation strategies and key activities under the national TB program in future should then accordingly target these factors. In future, we also propose looking into possible validation of these factors into a predictive tool to guide measures for risk assessment and stratification of TB patients.

## Conclusions

Our findings concluded that various sociodemographic characteristics as well as TB disease-related variables were associated with unsuccessful treatment TB outcomes and all-cause mortality. Together, these factors can guide measures for risk assessment and stratification of TB patients and subsequently, identify appropriate strategies of surveillance and management. Findings from our study also provide feedback to existing national TB control program and highlight areas to be focused on in future.
